# Intraventricular Tuberculoma with Profound Visual Loss: A Case Report and Literature Review

**DOI:** 10.7759/cureus.2807

**Published:** 2018-06-14

**Authors:** Amir Tengku-Fatishah, Alwi Muhd Besari, John Tharakan, Ismail Shatriah

**Affiliations:** 1 Ophthalmology, School of Medical Sciences, Universiti Sains Malaysia, 16150 Kubang Kerian, Kelantan, USA; 2 Medicine, School of Medical Sciences, Universiti Sains Malaysia, 16150 Kubang Kerian, Kelantan, MYS; 3 Neurosciences, School of Medical Sciences, Universiti Sains Malaysia, 16150 Kubang Kerian, Kelantan, MYS; 4 Ophthalmology, School of Medical Sciences, Universiti Sains Malaysia, 16150 Kubang Kerian, Kelantan, MYS

**Keywords:** tuberculosis, intraventricular tuberculoma, visual loss

## Abstract

Intracranial tuberculoma is a rare manifestation of tuberculosis involving the central nervous system. The involvement of the ventricular system is extremely uncommon. We describe a young woman with bilateral papilledema secondary to intraventricular tuberculoma with hydrocephalus. She was treated with anti-tuberculosis therapy and intravenous dexamethasone. Her visual acuity deteriorated after one month of treatment. We provide a literature review of this uncommon ocular sequelae.

## Introduction

Intracranial tuberculoma is a rare manifestation of tuberculosis. Recent data showed that cerebral tuberculoma accounted for 39%-46% of intracranial space occupying lesions [1-2]. It can spread to various portions of the brain and is commonly found in the cerebellum, basal ganglia, and cerebral hemisphere [3]. We report a rare case of intraventricular tuberculoma with hydrocephalus in a young woman who developed profound visual loss during the anti-tuberculosis treatment.

## Case presentation

A 27-year-old woman presented with a progressive painless visual loss in both eyes for one week prior to consultation. It was associated with preceding headache, nausea, and vomiting of two months duration. There was no history of prolonged fever, chronic cough, reduced weight, or loss of appetite. However, her father was treated for pulmonary tuberculosis two years ago and he had completed his anti-tuberculosis therapy.

**Figure 1:**
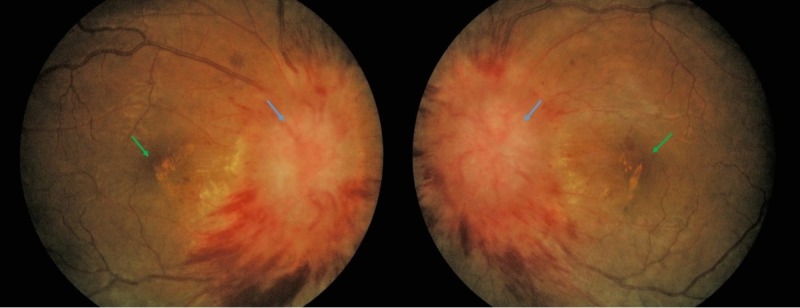
Fundus photography of bilateral eyes Bilateral papilledema with flame-shaped hemorrhages (blue arrow) and macular exudates (green arrow)

Her best corrected visual acuity was 3/60 (OD) and 4/60 (OS). The optic nerve function tests were impaired bilaterally and included light brightness, contrast sensitivity, and color vision; however, the right sight was more affected than the left. There was a relative afferent pupillary defect presence on her right eye. The confrontation test revealed generalized haziness with dense central scotoma on her bilateral vision. The anterior segment examination and intraocular pressure were essentially normal. The fundoscopy examination revealed bilateral papilledema. The optic discs were swollen and elevated with peripapillary flame-shaped hemorrhages as well as the presence of macular exudates (Figure 1). However, no sign of vitritis or panuveitis was observed.

On presentation, she was alert and orientated to time, place, and person. Her vital signs were stable. She was also afebrile. There were no signs of meningism or localizing signs. A respiratory examination revealed crepitation in the right lung. The other cranial nerves examinations were normal. The rest of the systemic examinations were unremarkable.

**Figure 2:**
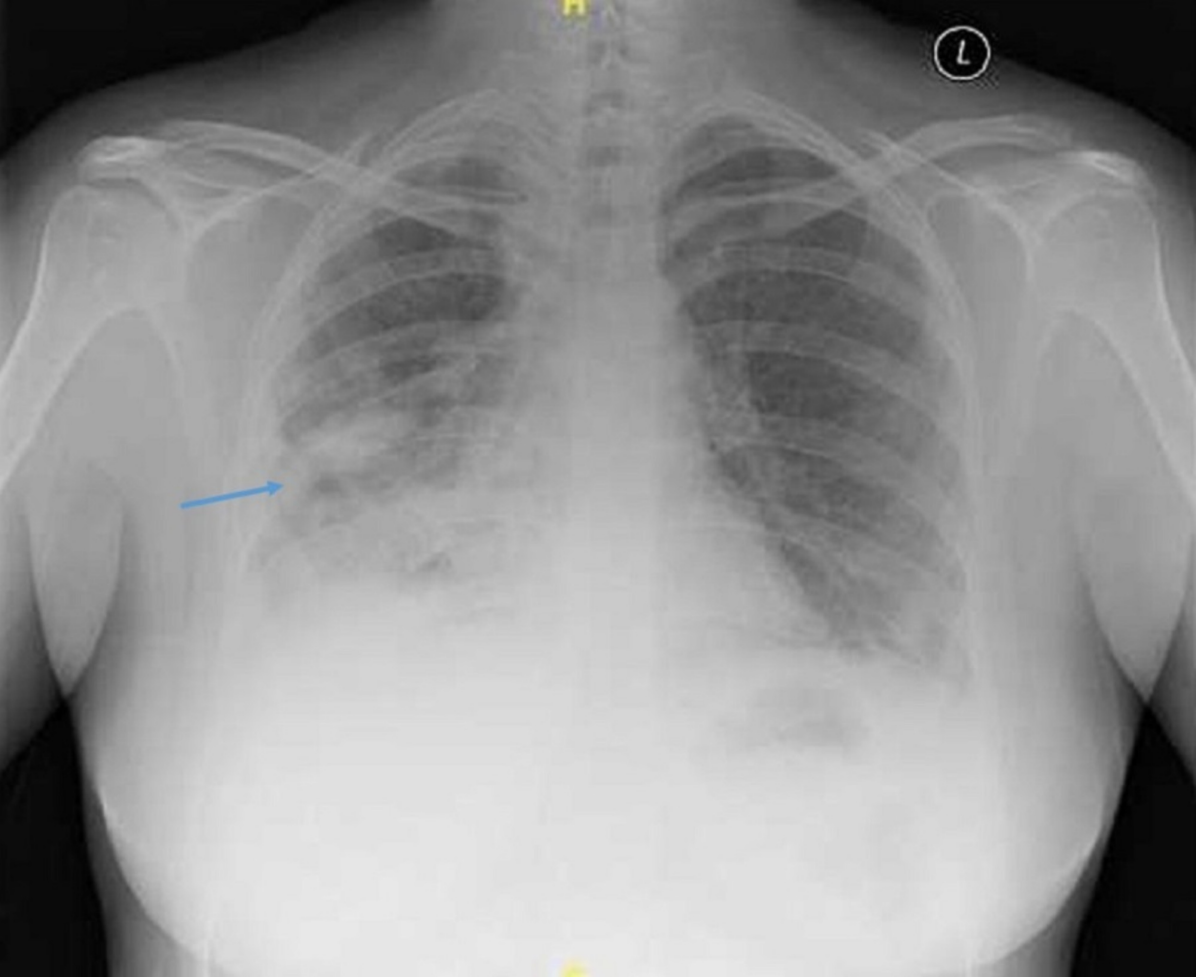
Chest radiograph Cavitation in the right lower zone (blue arrow)

Her baseline blood investigations were unremarkable except for an increased erythrocyte sedimentation rate (79 mm/h) and C-reactive protein (23 mg/l). The human immunodeficiency virus antibody test was negative. The Mantoux test was positive (16 mm area of induration). The sputum microscopic examination detected the presence of acid-fast bacilli. The chest radiograph showed cavitations at the right lower zone (Figure 2).

**Figure 3:**
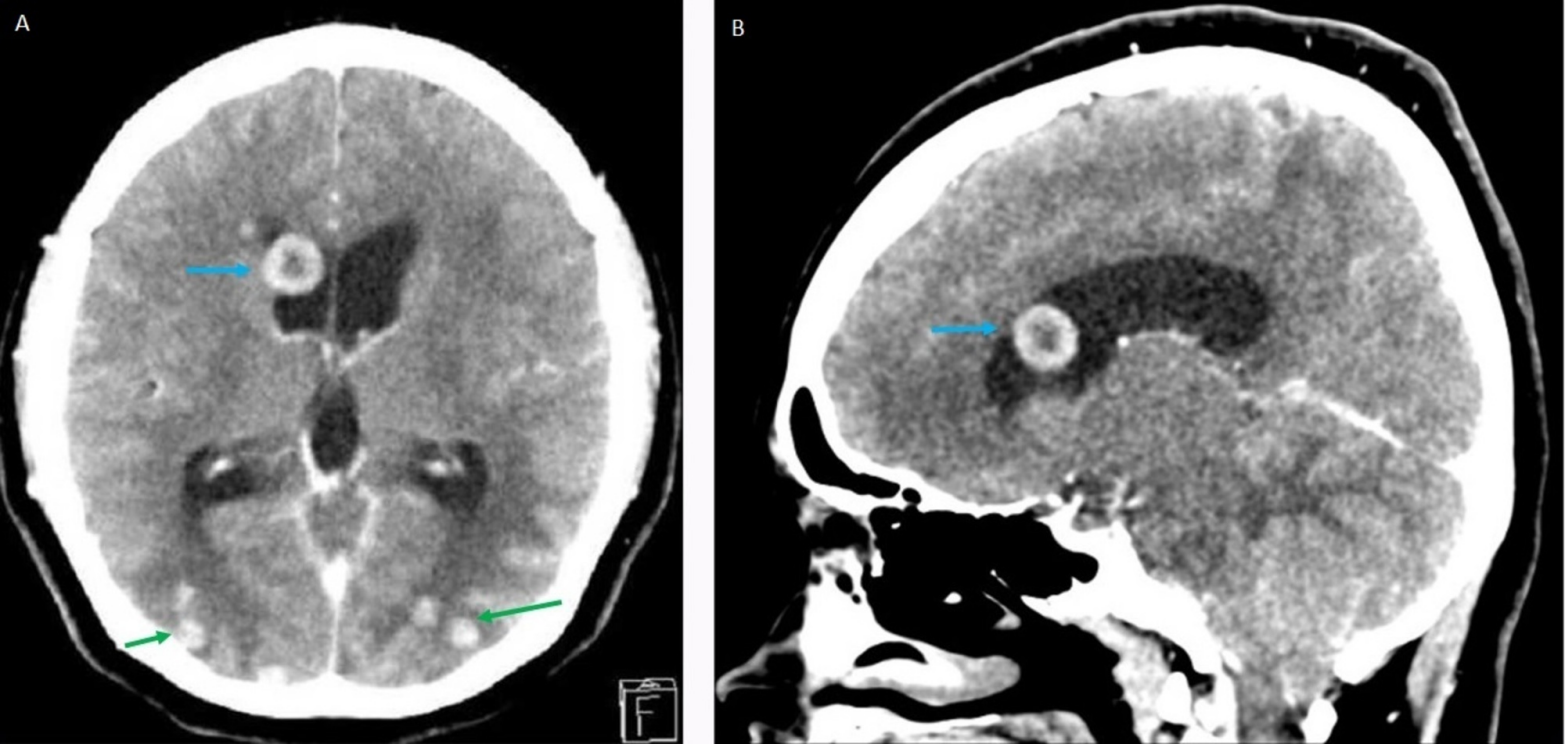
Computed tomography scan of the brain and orbit with contrast in the axial (3A) and sagittal cuts (3B) Solitary tuberculoma at the anterior horn of the right lateral ventricle (blue arrow). Multiple small parenchymatous discs were observed in the cerebrum (green arrow) with evidence of obstructive hydrocephalus

The computed tomography (CT) scan of the brain and orbit revealed the presence of multiple ring-enhancing hyperdense lesions with central hypodensity and perilesional edema. The lesions were located at the anterior horn of the right lateral ventricle (Figure 3A-3B). There were also multiple small lesions at the cerebrum and cerebellum (Figure 3A*)*.

However, there was no traction over the septum pellucidum. The lateral and third ventricles were dilated on both sides. She declined further diagnostic tests, including lumbar puncture and magnetic imaging resonance (MRI) of the brain and orbit. A presumptive diagnosis of bilateral papilledema secondary to intraventricular tuberculoma with hydrocephalus was considered. However, the patient refused the ventriculoperitoneal shunt procedure.

Anti-tuberculosis therapy was commenced immediately with oral isoniazid 300 mg, oral rifampicin 600 mg, oral pyrazinamide 1500 mg, intramuscular streptomycin 1000 mg, and intravenous dexamethasone 4 mg. Her symptoms of headache, nausea, and vomiting subsided.

**Figure 4:**
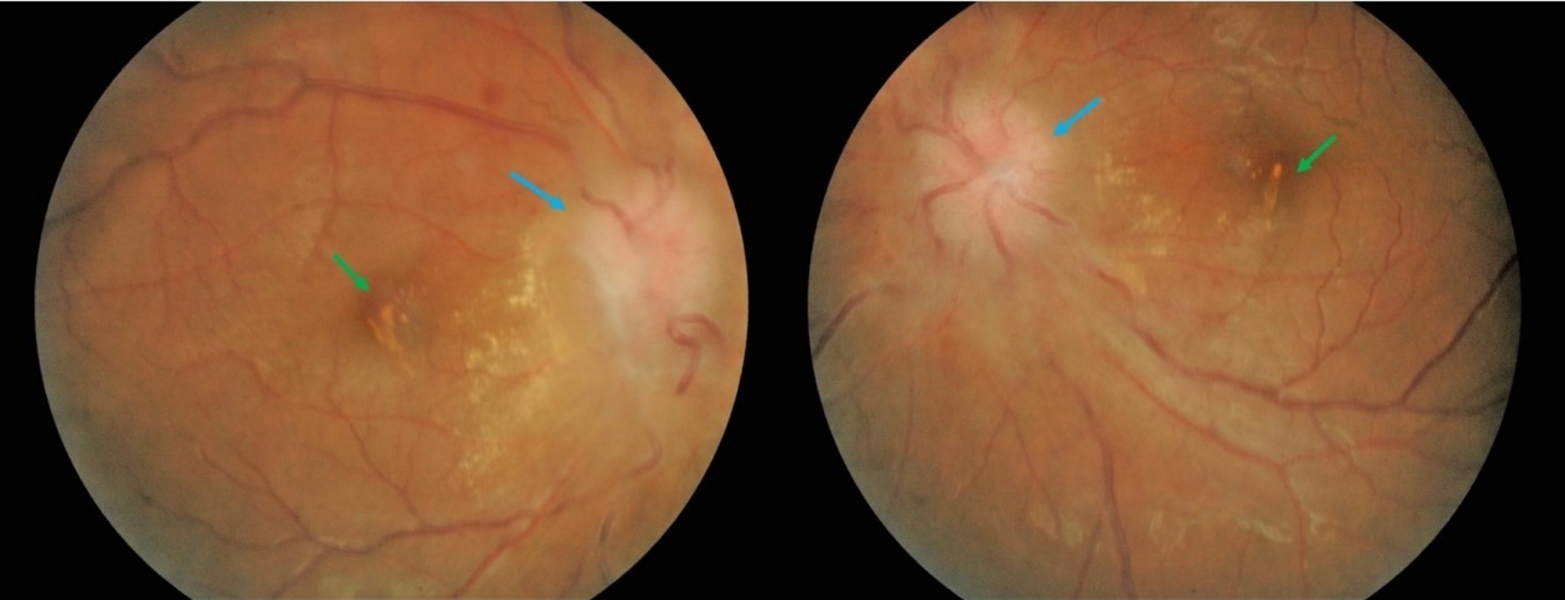
Fundus photography of bilateral eyes Bilateral pale optic disc (blue arrow) with regressing macular exudates (green arrows) after four weeks on anti-tuberculosis therapy

However, visual acuity of both eyes deteriorated progressively to non-perception of light after four weeks of treatment. She had no new emerging neurological symptoms or signs associated with the visual loss. A repeat funduscopy examination showed atrophy of the optic discs in both eyes (Figure 4).

We planned to extend her oral dexamethasone while continuing the same anti-tuberculosis regime. Unfortunately, the patient defaulted follow-up after one month of treatment.

## Discussion

A well-developed intraventricular tuberculoma is not common in tuberculosis infections. The effective blood circulation of the ventricular system acts as a barrier to infection despite evidence of tubercles in the choroid [3-6]. Based on the PubMed search, we encountered 18 published cases of intraventricular tuberculoma from 1987 to 2018 [3-14], including the current case report in Table 1. The age ranged from two years to 62 years. There were eight males and 11 females, including our patient.

**Table 1:** Published cases of interventricular tuberculoma from 1987 to 2018 VA, visual acuity; F, female; M, male; VP, ventriculoperitoneal

Author	Year	Sex/Age	Tuberculoma Location	Initial VA	Ocular Finding	Treatment	Follow-Up	Final Outcome
Berthier, et al. [3]	1987	M/4	Left lateral ventricle with traction of the septum pellucidum. Presence of asymmetrical hydrocephalus	Not available	Right oculomotor nerve palsy. No papilledema	Anti-tuberculosis	3 weeks	Good recovery
		F/5	Right lateral ventricle and right capsular region	Not available	Not available	Anti-tuberculosis	3 months	Good recovery
		M/4	Right lateral ventricle with traction of the septum pellucidum. Another lesion seen in the cerebral hemisphere. Presence of asymmetrical hydrocephalus	Not available	Bilateral papilledema with choroiditis	Anti-tuberculosis, Dexamethasone, VP shunt	Defaulted	After the VP shunt, the patient neurologically improved. However, he defaulted follow-up
		F/2	Left lateral ventricle, thalamus, cerebral, cerebellar, basal ganglia, and brainstem. Presence of hydrocephalus	Not available	No papilledema	Anti-tuberculosis	12 months	Good recovery
Vajramani, et al. [4]	1999	F/26	Right lateral ventricle. Presence of hydrocephalus	Not available	Bilateral sixth cranial nerve palsy. No papilledema	Anti-tuberculosis, Surgical excision, VP shunt	14 months	Good recovery
Desai, et al. [5]	2002	F/38	Within septum pellucidum. Presence of moderate obstructive hydrocephalus	Not available	No papilledema	Anti-tuberculosis, Surgical excision	9 months	Good recovery
Hsu, et al. [6]	2004	F/19	Right lateral ventricle. Presence of hydrocephalus	Not available	Mild papilledema	Anti-tuberculosis, Surgical excision, VP shunt	12 months	Good recovery
Sonmez, et al.[7]	2008	M/22	Right lateral ventricle. Presence of asymmetrical hydrocephalus	Not available	Not available	Surgical excision, Anti-tuberculosis	Not available	Not available
N’da, et al. [8]	2013	F/10	Third ventricle. Presence of obstructive hydrocephalus	Bilateral VA loss	Not available	Surgical excision, Anti-tuberculosis	6 months	Good recovery, however, final VA outcome was not mentioned
Coulibaly, et al. [9]	2013	M/26	Right lateral ventricle. Presence of asymmetrical hydrocephalus	OU 4/60	Not available	Surgical excision, External ventricular drainage, Anti-tuberculosis, Steroid	3 months	Total disappearance of the lesion. Final VA outcome was not mentioned
Udayakumaran, et al. [10]	2014	F/27	Third ventricle and thalamus. Presence of obstructive hydrocephalus	Not available	Not available	Anti-tuberculosis, Ofloxacin, Ethionamide, Endoscopic third ventriculostomy	12 months	Good recovery
Sachdeva, et al. [11]	2017	M/7	Foramen of Monro	Not available	Bilateral papilledema	Surgical excision, Anti-tuberculosis	15 months	Good recovery
Sharma, et al.[12]	2017	F/21	Third ventricle with obstructive hydrocephalus	Not available	Bilateral papilledema	VP shunt, Surgical excision, Anti-tuberculosis	1 month	Good recovery
Sadashiva, et al. [13]	2017	M/33	Left lateral ventricle with obstructive hydrocephalus	Not available	Not available	Anti-tuberculosis, Endoscopic biopsy, VP shunt	16 months	Good recovery
Li, et al. [14]	2017	F/62	Fourth ventricle	Not available	Not available	Intrathecal isoniazid, Dexamethasone, Anti-tuberculosis	9 months	Tuberculoma size decreased
		F/31	Fourth ventricle and right lateral ventricle	Not available	Not available	Intrathecal isoniazid, Dexamethasone, Anti-tuberculosis	3.5 months	Tuberculoma size decreased
		M/23	Right lateral ventricle	Not available	Not available	Intrathecal isoniazid, Dexamethasone, Anti-tuberculosis	7 months	Disappearance of tuberculoma
		M/47	Right lateral ventricle. Presence of hydrocephalus	Not available	Not available	Intrathecal isoniazid, Dexamethasone, Anti-tuberculosis	15 months	Disappearance of tuberculoma
Present study	2018	F/27	Right lateral ventricle, cerebral, and cerebellum. Presence of obstructive hydrocephalus	3/60 OD 4/60 OS	Bilateral papilledema	Anti-tuberculosis Dexamethasone	1 month	Bilaterally, no perception of light. Patient defaulted follow-up

The hematogenous spread of Mycobacterium tuberculosisthrough the choroidal plexus or ependyma is the common route of tubercle bacilli entry [3,5-7]. Berthier et al. reported that three of their patients had a lateral ventricle tuberculoma without choroidal plexus involvement [3]. These findings were similar to our patient who developed a right lateral ventricle tuberculoma without signs of choroidal plexus involvement. The tuberculoma in these patients probably arose from the subependymal tubercle.

Our patient developed a deterioration of visual acuity after four weeks of anti-tuberculosis therapy commencement. We postulate that this could be due to a paradoxical expansion of tuberculoma and the worsening of the pre-existing hydrocephalus. The paradoxical phenomenon has been described in multiple works of literature [14-19]. It is described as an excessive immune response by the host cell to the mycobacterial antigen that causes the expansion of the initial lesion or the growing of a new tuberculoma during the anti-tuberculosis therapy [17].

Another possible mechanism is due to poor drug penetration into the ventricular system. Udayakumaran et al. had performed an external third ventriculostomy in a case with a third intraventricular tuberculoma and obstructive hydrocephalus [10]. Despite multiple cerebral spinal fluid redirection procedures that had been performed along with the anti-tuberculosis regime, the tuberculoma was still being intractable. Thus, endoscopic third ventriculostomy was performed to excise the lesion and further cleared the cerebrospinal fluid route simultaneously [10]. Li and colleagues demonstrated a favorable outcome with the intrathecal isoniazid therapy [14].

A variety of treatment modalities had been attempted, as shown in Table 1. Five authors opted for prior surgical excision especially when the diagnosis was doubted [7-9,11-12]. This was due to the rarity of the tumor location or the absence of the systemic manifestation of tuberculosis infection. The lesion may also be imitating malignancy, as there were no specific clinical signs suggesting intraventricular tuberculoma. These patients were started on anti-tuberculosis treatment after a histopathology study confirmed tuberculoma [7-9,11].

Most of the published cases presented with features of hydrocephalus [3-10,12-14]. Six of these patients underwent cerebral spinal fluid redirection procedures, which included a ventriculoperitoneal shunt and external ventricular drainage [3-4,6,9,13-14]. Our patient was treated conservatively with an anti-tuberculosis regime and intravenous dexamethasone because she declined a ventriculoperitoneal shunt and further surgical intervention.

## Conclusions

Progressive visual loss during the treatment of an intraventricular tuberculoma with hydrocephalus is a challenging clinical scenario. Apart from poor drug penetration into the cerebrospinal fluid, the paradoxical reaction may be attributed to the recalcitrant nature of the disease. A standard guideline on the treatment regime is mandatory. This is very essential to prevent permanent visual loss and further neurological disability.
